# Should I stay, or should I go?

**DOI:** 10.36834/cmej.68161

**Published:** 2020-07-15

**Authors:** Sarah L. Silverberg

**Affiliations:** 1 University of British Columbia, British Columbia, Canada

Should I stay, or should I go?

It’s the question I’ve asked since I matched to residency. In fact, it has been on my mind long before then. Scenarios played out in my mind over and over during the clerkship, the R1 match, and my first year of residency.

The words of JK Rowling’s Albus Dumbledore echo in my mind: “…I sometimes think we sort too soon.” Like a pleuripotent stem cell, I could be happy in (almost) any field. Yet I found myself forced into a decision, bound by the pressure of the R1 match. How could I have explored the other specialties that had piqued my interest during clerkship, when I completed some after electives were booked? I could only apply to a few fields. I thought about withdrawing from the match process altogether since I was ripe with indecision. But the realities of our medical system—where entrance to residency is highly regulated and career changes once in practice are nearly impossible—made me back down from such a bold move. I was unwilling to risk losing the opportunity to start residency, when programs might think there is something wrong with me should I delay. In the end, I decided to enter the match and started residency in my first-choice discipline (out of the options I had left myself).

With the ratio of available residency seats to Canadian students applying to residency positions nearly 1:1 (or sometimes less), the opportunity to use electives as career exploration opportunities has become limited.^1^ Changes by the faculties to encourage parallel planning and electives in multiple disciplines may help.^2^ Yet with limited opportunities to transfer, the stakes of the R1 match remain impossibly high.^1^

Eight months into residency, I am still confused. I’ve had the privilege of a rotating internship—clerkship 2.0— since my program, like a handful of others, includes a basic clinical year. It has opened possibilities to consider other fields without the suffocating stress of the residency match pressuring me to pick one field and court it. For my peers, this year has re-affirmed their career decisions. For me, it has brought questions as to whether I should consider transferring to another program.

On my home program rotations, I have been torn between the satisfaction of having some confidence in the field, and alarm that I actually enjoy it. I was interested in some aspects of my work, and deeply disinterested by other parts. Week after week, my thoughts trailed into two streams: “I hate it here,” and, “I could never leave.” Like a fraught relationship, my emotions were torn between extremes.

I often felt like a fraud anytime our program got together. Could they see on my face that I hadn’t fully committed? Could they see my hesitation when making plans for next year’s rotations? I felt as though I was cheating on my co-residents with a tightly held secret.

Every time I become anxious regarding my residency, I try to figure out where I fit better, but I don’t quite fit into any of my alternatives either. I am too broad for the narrow fields, too narrow for the broad fields, too academic for the community-based practices, and too community for the tertiary care-based practices. I am enticed by other fields, but wonder if I risk believing that the grass will be greener on the other side. When I turn to staff physicians for advice, I feel as though they are using my indecision to validate their own career decisions.

Residents notoriously have high rates of burnout.^3^ The worry of being trapped in a discipline and the lack of career satisfaction that might stem from continuing in a discipline that doesn’t fit scares residents, myself included. Career choice regret in residents has found to be between 7.4% and 32.7%, varying by field, and symptoms of burnout have been associated with career and specialty choice regret.^4^ Many times this year, I have wondered whether my dissatisfaction has stemmed from symptoms of burnout, or whether it is true career regret.

I am told that I will find a way to be happy in any field. Others emphasize that residency is not a reflection of practice, and that career satisfaction increases later in one’s career.^5^ But what if what I want is not present in any of my options? What do you say to the resident who doesn’t quite fit anywhere?

My colleagues tell me I put too much pressure on myself and that I should see what I like. The trouble is each new rotation opens up opportunities that I had not fully considered. And when I disclose these feelings, I am told to stay put, since I cannot articulate why other options are better than my current choice.

What is hard to articulate is that it’s not just the clinical work I’m thinking about. It’s the career trajectory, the opportunities, and the question of how I can incorporate my other interests. If anything, residency is the worst time to be evaluating such things, when so little time is our own, and when our clinical responsibilities do not reflect the reality of practice.

Are these feelings common? Or am I alone in my uncertainty? Transfers are something that seem too taboo to discuss. And yet I can’t get it off my mind. I feel as though I cannot relate to my co-residents, seniors, or staff. My family doesn’t totally understand, and my friends are sick of hearing me think out loud.

While principles are in place to facilitate transfers, they are opaque, situational, and not well understood by residents. Efforts have only just begun at the national level to standardize the process.^6^ The lack of visibility of the process only increases anxiety for residents unsure of their future. Although the basic requirements for transfers are clear,^7^^,^^8^ how these principles are actually implemented is not.^3^ Some residents must complete electives during residency by carving time out of their core program, while others must remain in their program until the program is willing to release them. It feels like there is a black hole obscuring my view of what lies ahead.

The more I look around, the more I see established physicians who were once transfers themselves. They don’t publicize it, but it is a part of many peoples’ career development. According to Resident Doctors of Canada, about 3% of residents transfer, although up to one third will consider transferring to another program.^3^ These residents are often hidden, but once I started asking around, I found several other residents and staff who themselves had gone through the transfer process. Perhaps that will one day be me. I can only hope for that sincere of a decision. They all seem so happy; so certain.

What I am certain about is that I like medicine. I like talking to patients, thinking about complex problems, providing care. I like thinking about clinical problems in terms of the system that brings them about, and I like working on long-term solutions to better the care of populations.

I hope one day I will regain confidence in my path. I miss those days where the only choice was whether to choose medicine. I miss those days when the questions were simpler and the choices easier. They say it is about the journey and not the destination. But I am here wondering, should I embark on a new journey? Should I go, or should I stay?
